# Evolutionary Divergence and Biased Expression of NAC Transcription Factors in Hexaploid Bread Wheat (*Triticum aestivum* L.)

**DOI:** 10.3390/plants10020382

**Published:** 2021-02-17

**Authors:** Jianhui Ma, Meng Yuan, Bo Sun, Daijing Zhang, Jie Zhang, Chunxi Li, Yun Shao, Wei Liu, Lina Jiang

**Affiliations:** 1 College of Life Science, Henan Normal University, Xinxiang 453007, China; 2013033@htu.edu.cn (J.M.); YM140906@163.com (M.Y.); dxy2743056978@163.com (B.S.); 041160@htu.edu.cn (D.Z.); 443003@htu.edu.cn (C.L.); 041111@htu.edu.cn (Y.S.); 2 Collaborative Innovation Center of Henan Grain Crops, Agronomy College, Henan Agricultural University, Zhengzhou 450002, China; Zhangjie2935@163.com; 3 School of Life Sciences, Zhengzhou University, Zhengzhou 450001, China

**Keywords:** *Triticum aestivum*, hexaploidy, NAC transcription factors, evolutionary divergence, biased expression

## Abstract

The NAC genes, a large plant-specific family of transcription factors, regulate a wide range of pathways involved in development and response to biotic and abiotic stress. In this study, the NAC transcription factors were identified in 27 green plants, and the results showed that NAC transcription factors in plants undergo an appearance stage from water to land and a number expansion stage from gymnosperm to angiosperm. Investigating the evolutionary process of the NAC transcription factors from diploid species to hexaploid wheat revealed that tandem replications during the polyploidization process is an important event for increasing the number of NAC transcription factors in wheat. Then, the molecular characteristics, phylogenetic relationships, and expression patterns of 462 NAC transcription factors of hexaploid wheat (TaNACs) were analyzed. The protein structure results showed that TaNAC was relatively conservative at the N-terminal that contains five subdomains. All these TaNACs were divided into Group I and Group II by phylogenetic analysis, and the TaNACs in Group I should undergo strong artificial selection based on single nucleotide polymorphism (SNP) analysis. Through genome synteny and phylogenetic analysis, these TaNACs were classified into 88 groups and 9 clusters. The biased expression results of these TaNACs showed that there are 24 groups and 67 groups of neofunctionalization genes under biotic and abiotic stress, respectively, and 16 groups and 59 groups of subfunctionalization genes. This shows that neofunctionalization plays an important role in coping with different stresses. Our study provides new insights into the evolution of NAC transcription factors in hexaploid wheat.

## 1. Introduction

Transcription factors, known as trans-acting factors, could bind to specific DNA sequences in the promoter region of the target gene to activate or inhibit the transcription of associated genes. NAC is named after three gene fragments discovered in the 1970s, namely *NAM* (no apical meristem), *ATAF 1/2* (*Arabidopsis* transcription activator factor 1/2), and *CUC2* (cup-shaped cotyledon) [1,2,3]. It is a type of transcription factor specific to plants; it has multiple functions, and its proteins can usually be divided into two parts: the conserved N-terminal DNA-binding domain and highly differentiated C-terminal transcriptional regulatory regions [4,5,6,7]. The N-terminal can be further divided into five subdomains associated with DNA binding, dimer or heterodimer formation, and nuclear localization. The C-terminal is associated with activation/inhibition of transcription, and highly differentiated NAC proteins have a range of functions [6,8]. Many studies have reported that NAC transcription factors are involved in a wide range of processes, including plant growth and development, organ formation, signal transduction, and regulating the response to environmental stress [6,9,10,11,12].

In *Arabidopsis*, Ooka et al. and Nuruzzaman et al. identified 105 and 117 NAC transcription factors respectively. This provided important foundational data for functional investigation [4,13]. Mahmood et al. reported that *AtNAC032* could be induced in senescing leaves. Through a functional analysis, it could modulate reactive oxygen species production to positively regulate senescence in *Arabidopsis* [14]. Gladman et al. reported that *AtNAC78* and *AtNAC53* could bind to *cis*-elements of the 26S proteasome-stress regulon to mitigate proteotoxic stress [15]. Shahnejat-Bushehri et al. found one NAC transcription factor from *Arabidopsis*, JUB1, that forms the core to trigger DELLA accumulation, restricting cell elongation and further enhancing stress tolerance [16]. In rice, 151 non-redundant NAC transcription factors were identified to supplement the data of NAC transcription factors in rice [13]. Sakuraba et al. found that protein translated by *OsNAC106* could bind to *SGR*, *NYC1*, *OsNAC5,* and *LPA1* promoter regions to improve plant tolerance to senescence and salt stress [17]. The study of Shim et al. shows that overexpression of *OsNAC14* can improve the expression of genes related to stress response, DNA damage repair and defense, so as to improve drought resistance [18]. Sun et al. found that 63 OsNAC transcription factors were induced to be expressed under different abiotic (salt, drought, and cold) and biotic stresses (fungal, bacterial, viral pathogens, and parasitic plant infections) [19]. Therefore, NAC transcription factors are involved in multiple developmental processes and stress responses in a wide-range of plant species ranging from dicots to monocots.

Wheat is one of the typical poaceae plants; many studies have focused on the evolutionary analysis of NAC transcription factors in poaceae plants. NAC transcription factors were identified in barley [20,21]. The orthologous NAC transcription factors from *Brachypodium*, rice, *Arabidopsis* and barley showed sequence conservation and contain conserved functions through the analysis of HvNAC expression combined with functional verified NACs in other plants [20]. To further understand the evolution of NAC transcription factors, a phylogenetic characterization of NAC transcription factors from barley and other monocotyledonous plant species was performed and a promoter exchange was found that resulted in grain specificity of NAC-d-9 subgroup [21]. The NAC transcription factors were identified in durum wheat and bread wheat [22,23], and the homologous NAC transcription factors were got, which showed similar expressed pattern through the expression analysis using RNA-seq data. From the previous studies, we found that the orthologous NAC transcription factors in different species and homologous NAC transcription factors in one specie contain conserved functions in poaceae plants. However, the divergent functions of orthologous and homologous genes in poaceae plants were also observed by researchers [24,25,26]. In addition, the event of functional divergence was supposed to be an important impetus for plant evolution [27].

Wheat is a hexaploid plant and is one of the three major grain crops. Recently, the International Wheat Genome Sequencing Consortium released a high-quality wheat genome assembly version (Chinese Spring), which makes wheat gene research more accurate and reliable [28]. As a large family of transcription factors, the NAC transcription factors play a key role in regulating the development and stress response in wheat [9,23]. Uauy et al. identified a NAC transcription factor (NAM-B1) that regulates senescence and zinc and iron content in wheat [9]. Overexpression of *TaNAC47* in *Arabidopsis* could activate the expression of downstream genes to enhance the tolerance of transgenic plants to drought, salt, and freezing stress, and to combine with the ABRE *cis*-element to cause ABA allergy. Zhao et al. discovered a NAC transcription factor (*TaNAC-S*) that was mainly expressed in the leaf/sheath tissues and plays a regulatory role in leaf aging. Overexpression of *TaNAC-S* in wheat plants resulted in a delay in leaf aging, which may lead not only to an increased grain yield, but also an increase in the grain protein concentration [29]. Feng et al. isolated *TaNAC21/22* and found that this transcription factor plays a regulatory role in stripe rust as a target gene of tae-miR164 [30]. As many important TaNACs are functionally verified, it is of great significance to further study the NAC transcription factor family during the polyploidization process. In this study, the NAC transcription factors hexaploid wheat were identified. In addition, the polyploidization process and functional divergence of NAC transcription factor of hexaploid wheat was further analyzed, laying the foundation for further study of wheat NAC transcription factor.

## 2. Results

### 2.1. Identification of NAC Transcription Factors in Green Plants

To identify the NAC transcription factors in green plants, we performed an identification of NAC transcription factors in hexaploid wheat (AABBDD), *Triticum urartu* (AA), *Aegilops tauschii* (DD), *Triticum turgidum* (AABB), and 23 other green plants with the published genome-data. The 27 species represented eight plant evolutionary lineages, including green algae (*Botryococcusbraunii*, *Micromonas pusilla*, *Ostreococcus lucimarinus*, and *Volvox carteri*), a bryophyte (*Physcomitrella patens*), a lycophyte (*Selaginella moellendorffii*), a pteridophyte (*Azolla filiculoides*), two gymnosperms (*Gnetum montanum* and *Picea abies*), a basal angiosperm (*Amborella trichopoda*), monocotyledons (*Brachypodium distachyon*, *Oryza sativa*, *Sorghum bicolor*, *Zea mays*, *Triticum urartu*, *Aegilops tauschii*, *Triticum turgidum*, and *Triticum aestivum*), and dicotyledons (*Vitis vinifera*, *Eucalyptus grandis*, *Glycine max*, *Populus trichocarpa*, *Carica papaya*, *Arabidopsis thaliana*, *Theobroma cacao*, *Gossypium raimondii*, *Gsssypiium arboreu*, and *Popolus trichocarpa*). Among these 27 species, the NAC transcription factors in nine species have been identified. For identifying the NAC transcription factors in other species, local blast program was performed and the candidate NAC proteins from these genomes were verified using the Pfam protein family databases to search the conserve NAM domain (PF 02365). The number of NAC transcription factors and origin are provided in Supplementary Materials Table S1. The results showed that the NAC transcription factors were first identified in the bryophyte (*Physcomitrella patens*), indicating that these genes appeared as plants transitioned from water to land. The number of NAC transcription factors in bryophytes, lycophytes, pteridophytes, and gymnosperms was all less than 50, and increased to more than 70 and 100 in angiosperms. The results indicated that the NAC transcription factors underwent two important stages during green plants’ evolution. The first stage was the appearance of NAC transcription factors in green plants from water to land, and the second stage showed the expansion of NAC transcription factor number from gymnosperms to angiosperm.

### 2.2. Genome-Wide Identification of the NAC Transcription Factors from T. aestivum

All the high-confidence protein sequences were performed for HMM searching using the HMM profile from Pfam platform, and the gene family results are provided in Table S2. Based on HMM searching, 462 high-confidence TaNACs were identified (Table S3), including 158 sequences belonging to subgenome A, 150 sequences belonging to subgenome B, 145 sequences belonging to subgenome D, and nine sequences not located on chromosomes. The total length, molecular weight, and isoelectric point of NAC transcription factors were 104–1017 bp, 12.02–109.57 kda, and 4.31–10.50, respectively. The subcellular localization of each TaNAC was predicted using the platform of Plant-mPLoc and provided in Table S3. A nuclear localization signal (NLS) was detected in 453 TaNACs, a chloroplast localization signal was detected in four TaNACs, and subcellular localization signal could not be detected in five TaNACs. The annotation of these TaNACs and ID conversion among different genome versions of Chinese Spring are provided in Table S3 [28,31,32].

**Figure 1:**
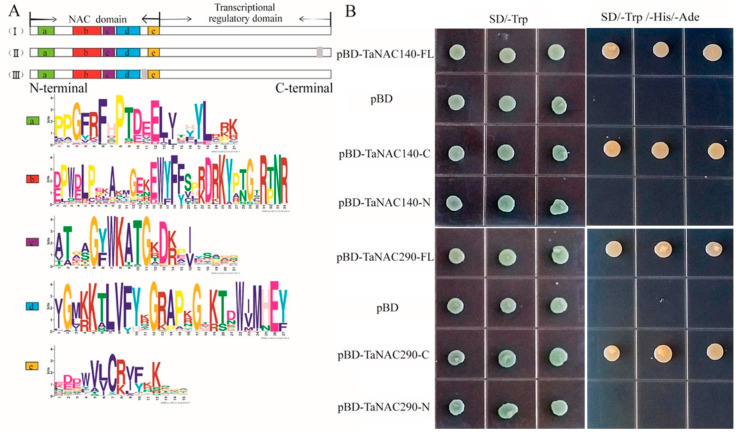
The protein structure (**A**) and transactivation activity assay (**B**) of TaNACs. (**A**) A schematic representation of NAC proteins: (**I**) Typical NAC protein with a highly conserved NAC domain at the N-terminal and transcriptional regulatory domain at C-terminal; (**II**) 19 membrane-bound TaNAC proteins with a transmembrane motif (gray square) at the C-terminal; and (**III**) one membrane-bound TaNAC protein with a transmembrane motif (gray square) between subdomains. The conserved NAC domain contains five subdomains, represented by some conserved amino acids (**a**–**e**). (**B**) The yeast trains containing pGBKT7-FL, pGBKT7-NAC-N, pGBKT7-NAC-C, and the negative control (pBD) grow on SD medium in the absence of tryptophan (SD/-Trp, first column) and in the absence of tryptophan, histidine, and adenine (SD/-Trp –His-Ade, second column). (FL: Full length; C: C-terminal; N: N-terminal).

The structure of TaNAC proteins was analyzed using the MEME platform. The results showed that TaNACs mainly contained a transcriptional regulatory domain at the C-terminal and a conserved NAC domain at the N-terminal (Figure 1A(I)), and the conserved NAC domain includes five subdomains that were presented by some conserved amino acids (Figure 1A(a–e)). To confirm this prediction, the NAC transcription factors (TaNAC140, and TaNAC290) were randomly selected for transactivation activity analysis (Figure 1B). The yeast trains contain pGBKT7-TaNAC, pGBKT7-NAC-domain, pGBKT7-NAC-C-ternimal and negative control (pGBKT7) grow well on SD medium in the absence of tryptophan (SD/-Trp), indicating a successful transformation (Figure 1B). The yeast trains containing pGBKT7-TaNAC and pGBKT7-NAC-C-terminal grow well on SD medium in the absence of tryptophan, histidine, and adenine (SD/-Trp –His-Ade), whereas the yeast trains containing pGBKT7-NAC-domain and negative control do not grow (Figure 1B). The results indicate that the C-terminal of TaNACs contains the transcriptional regulatory domain. Membrane-bound NAC proteins were predicted using the platform of TMHMM Server v.2.0, and the NAC proteins with a transmembrane motif were identified as membrane-bound NAC proteins. A total of 20 membrane-bound TaNAC proteins with one transmembrane motif were identified (Red color in Table S3), 19 of which were located on the outside of the conserved domain at the C-terminal (Figure 1A(II)), and the transmembrane motif of one sequence (*TraesCS2D01G378800*) located between the subdomains at the N-terminal (Figure 1A(III)).

**Figure 2:**
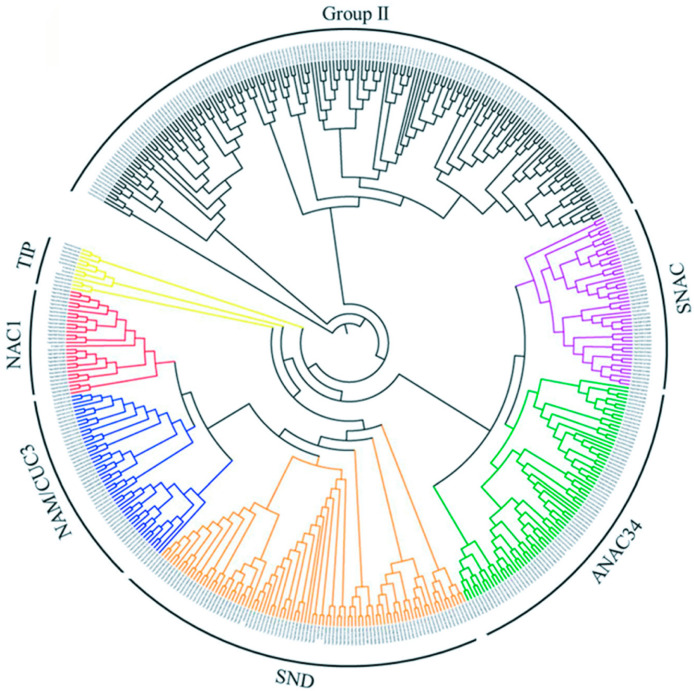
Phylogenetic tree analysis of TaNACs. An un-root phylogenetic tree was constructed using TaNACs and 13 AtNACs. TaNACs were divided into Group I and Group II. Group I was further divided into SNAC, ANAC34, SND, NAC1, NAM/CUC3, and TIP by the known AtNAC proteins.

To clarify the phylogenetic relationships of these TaNACs, a combined phylogenetic tree was constructed using the TaNAC proteins and 13 NAC proteins from *Arabidopsis* for the subgroup classification that was used in the previous study [13]. The results revealed that the TaNACs mainly fall into two major groups (Group I and Group II), and Group I was further divided into six subgroups, which were named by the known AtNAC proteins in each subgroup including SNAC (Stress-associated NAC), ANAC34, SND, NAC1, NAM/CUC3, and TIP (*Turnip crinkle* virus interacting protein). Group I consisted of 294 TaNACs; 43 belonged to SNAC, 76 to ANAC34, 87 to SND, 28 to NAC1, 50 to NAM/CUC3, and 10 to TIP; and Group II consisted of 168 TaNACs. Seventeen membrane-bound TaNACs out of 20 were clustered into the TIP subgroup (Figure 2). There were 79 groups containing three TaNACs from subgenome A, subgenome B, and subgenome D, respectively, in the phylogenetic tree, representing 51.30% of the total TaNACs.

### 2.3. The Evolutionary Process of the NAC Transcription Factors from Diploid Species to Hexaploid Wheat

Hexaploidy wheat (*Triticum aestivum*; AABBDD) is generated from the hybridization of *Aegilops tauschii* (DD) with *Triticum turgidum* (AABB). *Triticum urartu* (AA) is the subgenome A progenitor of *Triticum turgidum*. To investigate the evolutionary process of the NAC transcription factors from diploid species to hexaploid wheat, the NAC transcription factors from *Triticum urartu*, *Aegilops tauschii,* and *Triticum turgidum* were also identified from the published genome data [33,34,35]. One-hundred-and-twelve, 137 and 243 NAC transcription factors were identified in *Triticum urartu*, *Aegilops tauschii* and *Triticum turgidum*, respectively. Genome synteny and phylogenetic tree analysis were performed for these NAC transcription factors between species of hexaploidy wheat, *Triticum turgidum*, *Aegilops tauschii,* and *Triticum urartu*. From the results, we found that the NAC transcription factors in these species showed a strong evolutionary relationship (Figures S1–S7). Among the 112 NAC transcription factors in *Triticum urartu*, 81 showed a close evolutionary relationship with the NAC transcription factors in subgenome A of *Triticum turgidum*. Among the 137 NAC transcription factors in *Aegilops tauschii*, 111 showed a close evolutionary relationship with the NAC transcription factors in subgenome D of bread wheat. Among 121 NAC transcription factors from subgenome A and 117 NAC transcription factors from subgenome B in *Triticum turgidum*, 104 and 99 showed a close evolutionary relationship with the NAC transcription factors in subgenome A and subgenome B of bread wheat, respectively. From the above results, we found three types of evolutionary relationship, including a one-to-one relationship, a cluster-to-cluster relationship, and a one-to-cluster relationship. Most events of the one-to-cluster relationship were found during the process of diploid-to-tetraploid-to-hexaploidy or tetraploid-to-hexaploidy. So, tandem replications during polyploidization process are important for the number increasing of NAC transcription factors in wheat.

### 2.4. Genome Synteny Analysis of TaNACs

**Figure 3:**
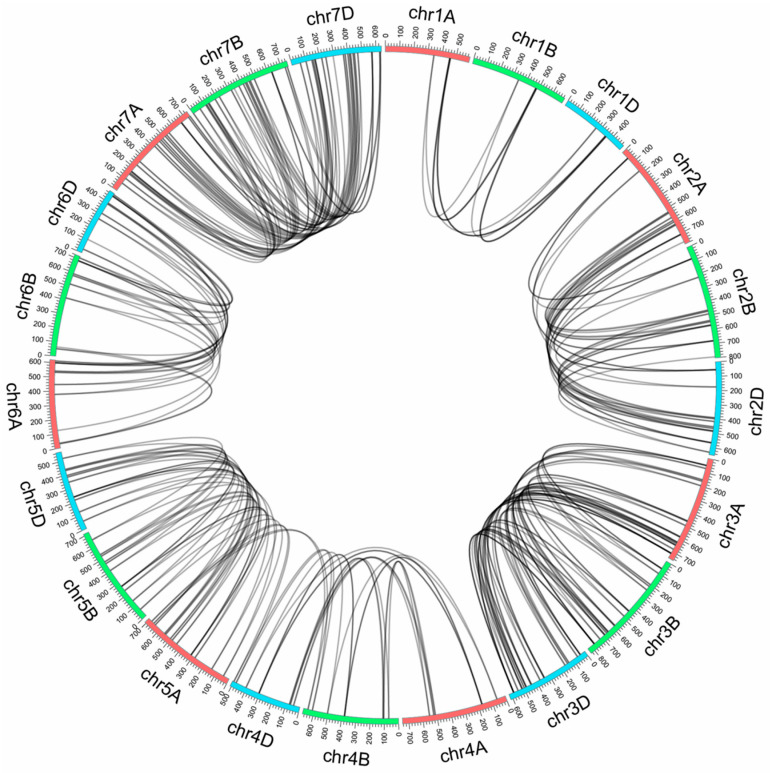
Circos diagram of the 21 chromosomes of bread wheat. The red ideograms represent the chromosomes from subgenome A, the green ideograms represent the chromosomes from subgenome B, and the blue ideograms represent the chromosomes from subgenome D. Putative homologous TaNACs are connected by black lines.

Genome synteny analysis is important for deciphering a genome’s evolutionary history [36]. The location of each TaNAC and homologous TaNACs on a genome was uncovered in the analysis (Figure 3). A synteny analysis of TaNACs from chromosome 1 to chromosome 7 is provided in Figure S8 to better understand the homologous relationship of TaNACs among homologous chromosomes. By genome synteny and phylogenetic tree analysis, 385 TaNACs were found to be in close relationship. Three TaNACs, which were clustered together and located on homologous chromosomes from subgenome A, subgenome B and subgenome D, respectively, were considered to be a group, and a total of 264 TaNACs were divided into 88 groups (Table S4). More than four TaNACs, which were clustered together from three sub-genomes on the homologous chromosome, were considered to be a cluster, and there should be two or more TaNACs in each cluster from the same sub-genome. A total of 98 TaNACs were clustered into nine clusters. TaNACs that were clustered together from two subgenomes on the homologous chromosome, were considered to be a pair, and a total of 23 TaNACs were clustered into four pairs (Table S4). Based on the above analysis, two sequences with no chromosome location information (*TaNAC456* and *TaNAC457*) matched with the B and D subgenomes were speculated to be from subgenome A. Three sequences with no chromosome location information (*TaNAC458*, *TaNAC460*, and *TaNAC461*) were grouped with the A and subgenome D that may belong to subgenome B, and one sequence with no chromosome location (*TaNAC454*) was grouped with the subgenome A and B, so it might be a gene from subgenome D. Three are still three sequences with no chromosome location information.

### 2.5. Cis-Component and SNP Analysis of the NAC Family Genes in T. aestivum

**Figure 4:**
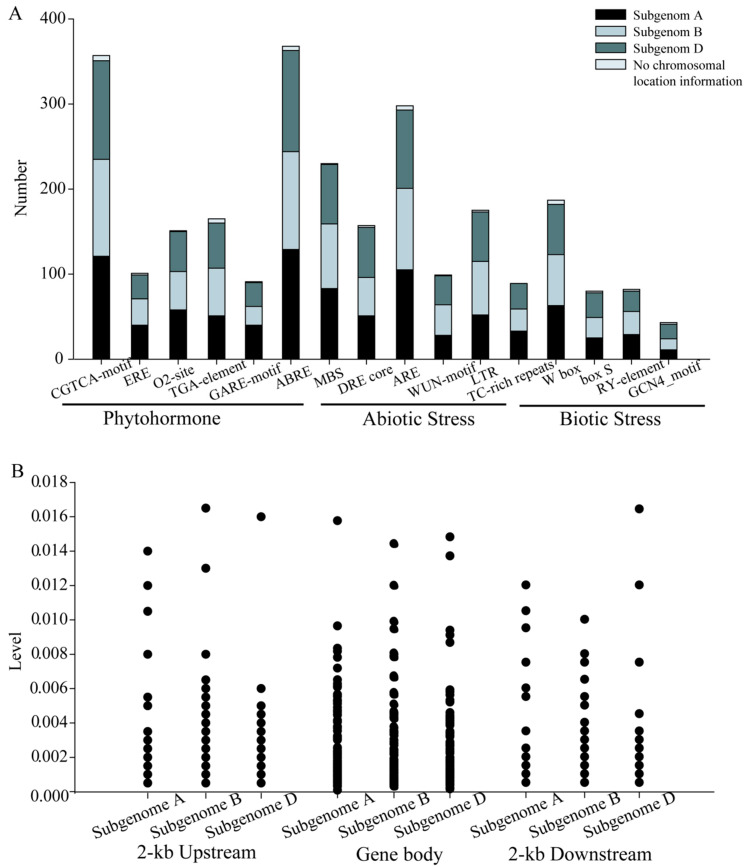
*Cis*-element (**A**) and SNP (**B**) analysis of the *TaNACs*. The 1.5-kb upstream sequences of the start codon of each *TaNAC* were used for *cis*-element analysis in the PLANTCARE database (**A**), and the *cis*-elements of *TaNACs* in each subgenome, including located on chromosomes, were counted. The gene body, 2-kb upstream and 2-kb downstream sequences of each *TaNACs* were selected for SNP density analysis in wheat populations (**B**), and each plot represented one *TaNAC* containing SNP.

*Cis*-element analysis is an effective method to study the potential of gene transcriptional regulation, hormone response, and biotic/abiotic stress response [12]. The promoter regions of *TaNACs* are abundant of regulatory elements (Figure 4A). Three *cis*-elements were found in more than 50% *TaNACs*, including abscisic acid response element (ABRE, 368 *TaNACs*), MeJA response element (CGTCA-motif, 357 *TaNACs*), and anaerobic induction element (ARE, 298 *TaNACs*). The cis-motifs of *TaNACs* associated with phytohormones and abiotic/biotic stress are abundant, including six cis-motifs involved in phytohormone response (CGTCA-motif, ERE, O2-site, TGA-element, GARE-element, and ABRE), four involved in biotic stress response (W box, box S, RY-element, and GCN4_motif), and six involved in abiotic stress response (MBS, DRE core, ARE, WUN-motif, LTR, and TC-rich repeats). The numbers of each *cis*-element distributed in each subgenome were not significantly different. This implied that *TaNACs* might play important roles in both the phytohormone response and stress response in wheat.

Bread wheat is the main cultivated crop worldwide with both natural selection and domestication playing important roles in its development. SNP density analysis is an important method to investigate molecular evolution. To investigate the genetic variation, we collected the re-sequencing data of hexaploid wheat plants [37]. The SNP density in the gene body, and in the 2-kb upstream and 2-kb downstream sequences of *TaNACs* of hexaploid wheat varieties, was calculated based on the re-sequencing data (Figure 4B). The average SNP density is 2.27 SNPs per kb, which is lower than the genome-wide level for wheat, with an average density of 4.18 SNPs per kb [38]. The number of TaNACs with SNP in a gene body is nearly twice than the number with SNP 2-kb upstream or 2-kb downstream. The average SNP density of TaNACs from Group II is 0.20% in the gene body, while it is just 0.09% of TaNACs from Group I. This indicates that the artificial selection of TaNACs in Group I should be stronger than that in Group II.

### 2.6. Biased Expression of TaNACs in Response to Abiotic Stress

As important regulators, NAC transcription factors had been found to be involved in different stress signaling [12,39,40]. Here, the published transcriptome data of *T. aestivum* treated with cold (SRP043554), heat (SRP045409), drought (SRP045409), and PEG (SRP145238) were used to investigate the expression patterns of TaNACs under abiotic stress [41,42,43]. The differentially expressed genes (DEGs) were identified by the parameters of 2-fold change and *p*-value < 0.05 between treatment and control. The groups that were identified through phylogenetic and genome synteny analysis were used for biased expression analysis. And the groups with the FPKM of all TaNACs < 1 were removed, and the remained groups, containing differentially expressed TaNACs, were analyzed. According to the parameters for DEGs selection, the up- and down-regulated TaNACs were identified under different treatments. Under abiotic stress, 140 differentially expressed TaNACs were up-regulated under at least one treatment, among which five differentially expressed TaNACs (*TaNAC19*, *TaNAC130*, *TaNAC251*, *TaNAC251*, and *TaNAC275*) were significantly up-regulated under the cold, heat, drought, and PEG treatments compared with the control. On the other hand, 112 differentially expressed TaNACs were down-regulated under at least one abiotic stress, and the two differentially expressed TaNACs (*TaNAC393* and *TaNAC440*) were down-regulated under four abiotic stresses. There were 32, 35, 72, and 83 up-regulated TaNACs and 39, 66, 25, and 25 down-regulated TaNACs under the cold, heat, drought, and PEG treatments, respectively.

**Figure 5:**
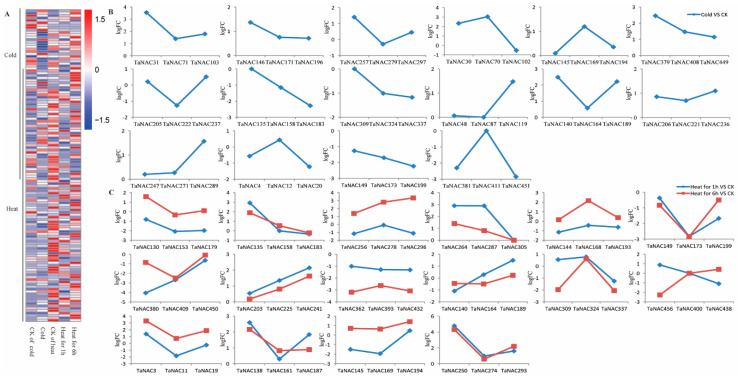
Heat map of expression profiles (FPKM) of differentially expressed *TaNACs* (**A**), and $log_{2}^{Fold\;change}$ (logFC) of TaNAC groups under low temperature stress (n = 3, (**B**)) and heat stress (n = 2, (**C**)) compared with CK. (**A**) Expression levels of differentially expressed *TaNACs* are present according to the color scale, in which red color and blue color represent high and low transcript abundance respectively. The line on the left means differentially expressed *TaNACs* under low temperature stress or heat stress, and the treatments are indicated at the bottom of each column. Three *TaNACs* with high homology in three subgenomes by phylogenetic and genome synteny analysis were clustered into one group, and the line represents the logFC of *TaNACs* under low temperature stress (**B**) and heat stress (**C**) compared with CK.

Under cold stress, the differentially expressed TaNACs mainly belonged to 25 groups (Figure 5A). The TaNACs in nine groups showed similar expressed pattern, indicating sub-functionalization of these TaNACs in each group. Among the nine groups, the expression level of TaNACs in four groups was significantly up-regulated and the expression level of TaNACs of five groups was down-regulated (Table S5). The TaNACs in the other 16 groups, including differentially expressed and non-differentially expressed TaNACs in each group, showed different expression patterns, indicating neofunctionalization of these TaNACs (Figure 5B). Under heat treatment, the differentially expressed TaNACs mainly belonged to 33 groups (Figure 5A). The TaNACs in 16 groups showed a similar expressed pattern, indicating sub-functionalization of these TaNACs in each group, and they were divided into two types according to expression pattern (Table S6). The TaNACs in 17 other groups, including differentially expressed and no-differentially expressed TaNACs in each group, showed different expression patterns, indicating neofunctionalization of these TaNACs (Figure 5C).

**Figure 6:**
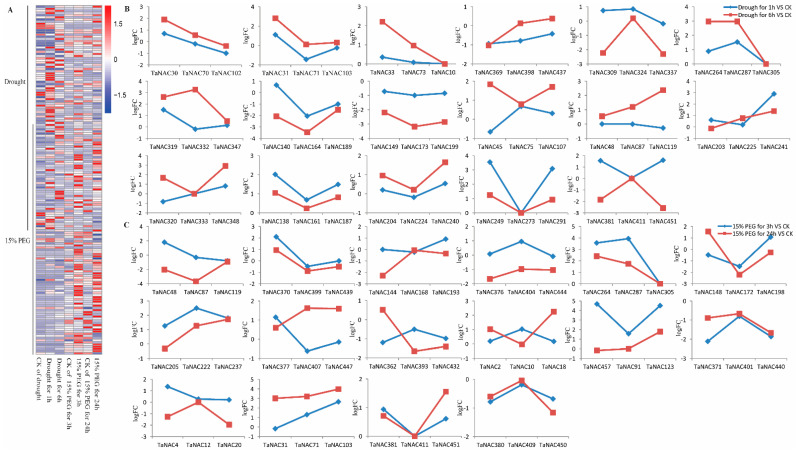
Heat map of expression profiles (FPKM) of differentially expressed *TaNACs* (**A**), and the $log_{2}^{Fold\;change}$ (logFC) of TaNAC groups under drought stress (n = 2, (**B**)) and osmotic stress (n = 3, (**C**)) compared with CK. (**A**) Expression levels of differentially expressed *TaNACs* are present according to the color scale, in which red color and blue color represent high and low transcript abundance respectively. The line on the left means differentially expressed *TaNACs* under drought stress or osmotic stress, and the treatments are indicated at the bottom of each column. Three *TaNACs* with high homology in three subgenomes by phylogenetic and genome synteny analysis were clustered into one group, and the line represents logFC of *TaNACs* under drought stress (**B**) and osmotic stress (**C**) compared with CK.

Under drought stress, the differentially expressed TaNACs mainly belonged to 31 groups (Figure 6A), among which the TaNACs in 14 groups showed sub-functionalization (Table S7) and the TaNACs in 17 groups showed neofunctionalization (Figure 6B). Under osmotic stress, the differentially expressed TaNACs mainly belonged to 37 groups (Figure 6A), among which the TaNACs in 20 groups showed sub-functionalization (Table S8); the TaNACs in 17 groups showed neofunctionalization (Figure 6C). Through the analysis of differentially expressed TaNACs under abiotic stress, it was found that neofunctionalization was the one way for TaNACs to deal with abiotic stress (Table S9).

### 2.7. Biased Expression of TaNACs in Response to Biotic Stress

In addition to regulating abiotic stress, NAC transcription factors were also found to be involved in biotic stress. The published effective RNA-seq data of wheat related to biotic stress (SRP041017) were downloaded [44], and the expression patterns of TaNACs under different biotic stresses were explored. Forty-seven and 51 differentially expressed TaNACs in response to powdery mildew and stripe rust stress were found, respectively. Under the powdery mildew treatment, 28 genes were up-regulated and 19 were down-regulated. Under stripe rust infection, there were 23 up-regulated TaNAC genes and 21 down-regulated TaNAC genes.

**Figure 7:**
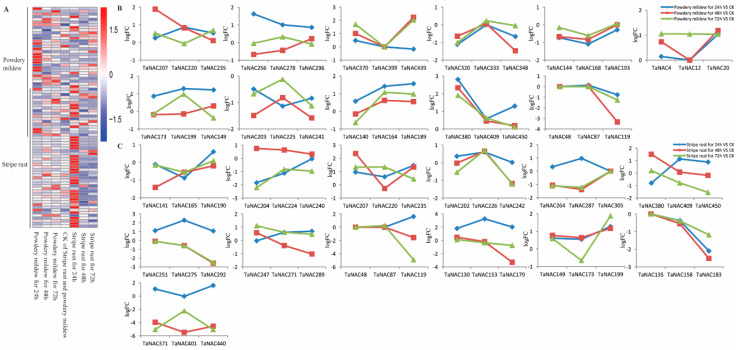
Heat map of expression profiles (FPKM) of differentially expressed *TaNACs* (**A**), and the $log_{2}^{Fold\;change}$ (logFC) of TaNAC groups under powdery mildew stress (n = 3, (**B**)) and stripe rust stress (n = 3, (**C**)) compared with CK. (**A**) Expression levels of differentially expressed *TaNACs* are presented according to the color scale, in which red color and blue color represent high and low transcript abundance respectively. The line on the left means differentially expressed *TaNACs* under powdery mildew stress or stripe rust stress, and the treatments are indicated at the bottom of each column. Three *TaNACs* with high homology in three subgenomes by phylogenetic and genome synteny analysis were clustered into one group, and the line represents the logFC of *TaNACs* under powdery mildew stress (**B**) and stripe rust stress (**C**) compared with CK.

The differentially expressed TaNACs and their group were screened under biotic stresses (Figure 7A). Under powdery infection, the differentially expressed TaNACs mainly belonged to 18 groups, among which the TaNACs in seven groups showed sub-functionalization (Table S10) and the TaNACs in 11 groups showed neofunctionalization (Figure 7B). Under stripe rust, the DEGs mainly belonged to 22 groups, among which the TaNACs in nine groups showed sub-functionalization (Table S11) and the TaNACs in 13 groups showed neofunctionalization (Figure 7C). Through the analysis of gene expression in groups under powdery/stripe rust stress, it was found that neofunctionalization was an important way for TaNACs to deal with biotic stress (Table S12).

## 3. Discussion

The NAC transcription factor family is one of the largest families and has been studied in a large number species [12,13,45,46]. The completion of genome sequence of various plants realized the identification and comparative analysis of NAC transcription factors in green plants. In this study, eight plant evolutionary lineages, including 27 types of green plants, were selected for the evolutionary analysis of NAC transcription factors. We found that NAC transcription factors were only in land plants, and number expansion from gymnosperms to angiosperms. This may be closely linked with stress response and plant development. Bread wheat is a hexaploidy species (AABBDD), and it is derived from the hybridization of *Aegilops tauschii* (DD) with *Triticum turgidum* (AABB). *Triticum urartu* (AA) is the subgenome A progenitor of *Triticum turgidum*, and the subgenome B progenitor of *Triticum turgidum* has not been ensured until now. This study focused on the evolutionary process of the NAC transcription factors from diploid species to hexaploid wheat. One-hundred-and-twelve, 137, 243, and 462 NAC transcription factors were identified in *Triticum urartu*, *Aegilops tauschii*, *Triticum turgidum*, and bread wheat, respectively. We found that the number of NAC transcription factors showed increased during the polyploidization process. From the evolutionary relationship of NAC transcription factors from diploid species to hexaploid wheat (Figures S1–S7), we found most single copy and clusters of NAC transcription factor were retained. However, the event of tandem replication was observed from some single copy of NAC transcription factor form diploid species to hexaploid wheat. It indicated that tandem replication for cluster formation during the polyploidization process is an important event for increasing the number of NAC transcription factors in wheat, which is consistent with the research in other species [47].

Due to the complexity and large genome of bread wheat, it should have more NAC transcription factors than other species, and many researchers have already performed research to identify TaNACs from the wheat genome [23,48]. Borrill et al., identified 454 TaNACs using the TGAC wheat assembly, and 146 homologous triads of TaNACs were found. In this research, 462 TaNACs were identified using IWGS RefSeq v1.0 database, among which 382 TaNACs were in common with the research of Borrill et al. (Table S3). As evidence of the high homology of wheat subgenomes, 101 homologous triads of TaNACs were found (Table S4), which is significantly less than the previous study [23]. In addition, 87% of triads had a single copy of each homology that is considered to be one group (88 groups), 9% of triads showed variable number that is considered to be one cluster (nine clusters), and 4% of triads had one or several homologs with one homolog absent that is considered to be one pair (four pairs). Compared with two researches, we found some homologous TaNACs with close tandem replication lost in Borrill’s research. Taking RefSeqv1.0 assembly as the gold standard, this study should provide more overall information about *TaNACs*.

A phylogenetic tree analysis of these TaNACs was performed, and the results showed that the TaNACs could be divided into two groups; Group I was further divided into six subgroups, which is consistent with the NAC transcription factor of rice and *Arabidopsis* [13]. It was found that almost TaNACs contained *cis*-elements related to abiotic and biotic stresses, and all the DEGs under four abiotic stresses contained at least one *cis*-element. Through SNP analysis, it was found that the TaNACs that were differentially expressed under abiotic or biotic stresses were more conserved than others, especially TaNAC250 and TaNAC251, of which the SNP densities were 0. This could be related to natural and/or artificial selection.

Similar expressed pattern of homologous NAC transcription factors was observed in durum wheat and bread wheat [22,23]. Due to the importance of functional divergence for plant evolution [27] and the important functions of TaNACs, we focused on the biased expression of *TaNACs* to understand the TaNAC evolution. By comparing the expression pattern of each TaNAC in one group, it was found that neofunctionalization was one of the important ways, whether under biotic stress or abiotic stress. There were still some selection phenomena for a subgenome under osmotic stress. It was found that the differentially expressed TaNACs preferred to select the subgenome A and subgenome D under the osmotic stress. Gene duplication is important event in the process of polyploidization in plants. In addition, the duplicate genes usually have divergent functions [49]. Neofunctionalization supports the notion that one gene maintains the ancestral function while the other gets new functions after duplication [50], and neofunctionalization is an important impetus for plant evolution [27]. The event of neofunctionalization had been detected in wheat and supposed to contribute to the adaptation of wheat to a diversity of conditions [25,26]. Bias expression of TaNACs in many homologous groups is observed in this study, indicating that neofunctionalization of TaNACs happens during evolution from a diploid species to hexaploid wheat. As an important function in grain yield, nutrition, and stresses [9,51,52], the neofunctionalization of TaNACs should play an important role in maintaining the hexaploid wheat as a global staple food.

## 4. Materials and Methods

### 4.1. Identification of NAC Transcription Factor from T. aestivum and Other Green Plants

To identify the NAC transcription factors from *T. aestivum* (TaNACs), protein sequences with high-confidence of *T. aestivum* were downloaded from the International Wheat Genome Sequencing Consortium [28]. All the sequences were used for Hidden Markov Model (HMM) searching (PF02365) with an E-value cut-off of 1 × 10^−5^ using the database from Pfam to identify the candidate TaNACs. The candidate TaNACs were used to compare with previous studies for TaNACs identification, and extra sequences were further confirmed by the Pfam and SMART platforms for TaNACs identification [23,48].

The previous studies had identified NAC transcription factor from Oryza sativa, Sorghum bicolor, Zea mays, Theobroma cacao, Glycine max, Gossypium raimondii, Gsssypiium arboretum, and Popolus trichocarpa [12,13,53,54,55,56,57]. The NAC transcription factors from Arabidopsis were confirmed in Tair [58]. For identification of NAC transcription factors from Triticum Urartu, Aegilops tauschii, and Triticum turgidum, the published genome data was download [35,59,60], and the TaNACs were used to perform Blastp. The homologous sequences were used for HMM searching (PF02365) with an E-value cut-off of 1 × 10^−5^ using the database from Pfam to identify NACs transcription factors. The genome data of Botryococcusbraunii, Micromonas pusilla, Ostreococcus lucimarinus, Volvox carteri, Physcomitrella patens, Selaginella moellendorffii, Azolla filiculoides, Gnetum montanum, Picea abies, Amborella trichopoda, Brachypodium distachyon, Vitis vinifera, Carica papaya, and Eucalyptus grandis were downloaded from Phytozome V12 [61], and the method for identification of NAC transcription factors from Triticum Urartu was used to identify NAC transcription factors in these species. The number, origin, and gene ID are provided in Table S1.

### 4.2. Cloning and Transactivation Activity Assay of TaNAC140, and TaNAC290

The sequence of *TaNAC140* and *TaNAC290* was obtained from the genome data [28], and specific primers were designed (Table S13, PF1, PR1, PF2, and PR2) for gene cloning. The two sequences were identified and were the same with genome data. The C-terminals and N-terminals of both sequences were predicted in PFAM database, and the primers were designed (Table S13, PF3, PR3, PF4, PR4, PF5, PR5, PF6, and PR6). The recombinant plasmids of pBD-FL, pBD-N, pBD-C, and pBD of two sequences were transformed into Y2H Gold Yeast (WEIDI, Beijing, China) respectively. All transformed yeasts were screened on SD/-T (Coolaber, Beijing, China). The successfully transformed yeast cells were transferred to SD/-T/-H/-A (Coolaber, Beijing, China). The yeasts were cultured in an incubator at 30 °C to evaluate the transactivation activity.

### 4.3. Gene Structure and Phylogenetic Analysis

All protein sequences of TaNACs were analyzed using Muscle and MEME to detect conserved motifs of NAC transcription factors [62]. The TMHMM Server v.2.0 was used to filter out the membrane-bound NAC proteins. Plant-mPLoc was used to predict the subcellular localization of each TaNAC [63].

For phylogenetic analysis, multiple alignment was performed using the full-length protein sequences of TaNACs by Muscle; the neighbor-joining method was used to construct the un-root phylogenetic trees using MEGA 5.10, and bootstrapping was set to be 1000 replicates to assess the significance of each node [64].

### 4.4. Chromosomal Location and Collinearity Analysis

For the collinearity analysis, gff3 annotation file containing gene locations information was downloaded from IWGSC and converted into a GFF file for further analysis. All protein sequences of hexaploid wheat were aligned using BLASTp with an E-value cut-off of 1 × 10^−5^. The aligned BLASTp result and GFF file were analyzed using MCScanX to calculate the collinear region of the entire genome [65]. Finally, the collinearity block containing the TaNACs was screened, and collinearity pairs were used to draw a collinearity map using CIRCOS [66].

### 4.5. Cis-Element Analysis

Location information of TaNACs was extracted from the annotation file. According to the retrieved location information, the 1.5 kb upstream sequences of the TaNACs initiation codon were intercepted, and the sequences were uploaded to PlantCARE for *cis*-component analysis [67].

### 4.6. Transcriptome Data and Gene Expression Pattern Analysis

Transcriptional data related to biotic/abiotic stresses of hexaploid wheat were downloaded from NCBI [38,39,40,41]. The downloaded sra files were converted into Fastq files using fastq-dump. HISAT2 (version: 2.1.0) was used for mapping reads. Then, the view ubroutine in samtools (version: 0.1.19) was used to filter out the nonunique mapping reads and mismatches. Finally, stringtie (version: 1.3.3b) was applied to analyze the data, and fragments per kilobase million (FPKM) value was used to represent the gene expression level [68].

## 5. Conclusions

This study investigated the evolution of NAC transcription factors in green plants and found that NAC transcription factors in plants undergo an appearance stage from water to land and a number expansion stage from gymnosperms to angiosperms. Analyzing the evolutionary process of the NAC transcription factors from diploid species to hexaploid wheat, we found that tandem replications contribute to the increase in the number of NAC transcription factors in wheat. The 462 TaNACs were classified into 88 groups, 9 clusters and 4 pairs through phylogenetic and genome synteny analysis. Molecular characterization and transactivation activity analysis showed that the C-terminal of TaNACs contains the transcriptional activity. Through a phylogenetic analysis, all these TaNACs were divided into Group I and Group II; the TaNACs of Group I can be further subdivided into six groups. Variation analysis of *TaNACs* showed that *TaNACs* have a lower mutation rate compared with genome wheat [38], and a *cis*-acting analysis of *TaNACs* indicated that TaNACs play important roles in both phytohormone and stress responses in wheat. Expression level analysis under different stress revealed that there are more TaNAC groups with neofunctionalization than groups with sub-functionalization, which showed that neofunctionalization is an important way for TaNACs to deal with stress. Our results provide a new perspective on the evolution of NAC transcription factors in hexaploid bread wheat.

## Data Availability

The data presented in this study are available in Supplementary Materials.

## Supplementary Materials

The following are available online at https://www.mdpi.com/2223-7747/10/2/382/s1. Figure S1. The evolutionary relationship of NAC transcription factors in Chr 1 between *Triticum urartu* (Tu)*, Aegilops tauschii* (At), *Triticum turgidum* (Tt) and hexaploid wheat (Ta). Figure S2. The evolutionary relationship of NAC transcription factors in Chr 2 between *Triticum urartu* (Tu)*, Aegilops tauschii* (At), *Triticum turgidum* (Tt) and hexaploid wheat (Ta). Figure S3. The evolutionary relationship of NAC transcription factors in Chr 3 between *Triticum urartu* (Tu)*, Aegilops tauschii* (At), *Triticum turgidum* (Tt) and hexaploid wheat (Ta). Figure S4. The evolutionary relationship of NAC transcription factors in Chr 4 between *Triticum urartu* (Tu)*, Aegilops tauschii* (At), *Triticum turgidum* (Tt) and hexaploid wheat (Ta). Figure S5. The evolutionary relationship of NAC transcription factors in Chr 5 between *Triticum urartu* (Tu)*, Aegilops tauschii* (At), *Triticum turgidum* (Tt) and hexaploid wheat (Ta). Figure S6. The evolutionary relationship of NAC transcription factors in Chr 6 between *Triticum urartu* (Tu)*, Aegilops tauschii* (At), *Triticum turgidum* (Tt) and hexaploid wheat (Ta). Figure S7. The evolutionary relationship of NAC transcription factors in Chr 7 between *Triticum urartu* (Tu)*, Aegilops tauschii* (At), *Triticum turgidum* (Tt) and hexaploid wheat (Ta). Figure S8. Circos diagram of TaNACs from chromosome 1 to chromosome 7 of bread wheat. Table S1 The ID and origin of NAC transcription factors in different species. Table S2. The results of HMM searching of high-confidence protein sequences of wheat genome. Table S3. The annotations of TaNACs and ID conversion among different genome versions of Chinese Spring. Table S4. TaNACs belonged to 88 groups, nine clusters and four pairs. Table S5. Sub-functionalization of TaNACs under cold stress. Table S6. Sub-functionalization of TaNACs under heat stress. Table S7. Sub-functionalization of TaNACs under drought stress. Table S8. Sub-functionalization of TaNACs under osmotic stress. Table S9. Bias expression of TaNACs under abiotic stress. Table S10. Sub-functionalization of TaNACs under powdery stress. Table S11. Sub-functionalization of TaNACs under stripe stress. Table S12. Bias expression of TaNACs under biotic stress. Table S13. The primers used in this study.
