# Supplementary material for: Evolutionary Divergence and Biased Expression of NAC Transcription Factors in Hexaploid Bread Wheat (Triticum aestivum L.)
Source: Plants (Basel). 2021 Feb 17;10(2):382. doi: 10.3390/plants10020382 (PMC7922369; doi:10.3390/plants10020382)
Supplement: Supplementary file 1 [file plants-10-00382-s001.zip › Supplementary Files/Figure S1-S8.docx]

**
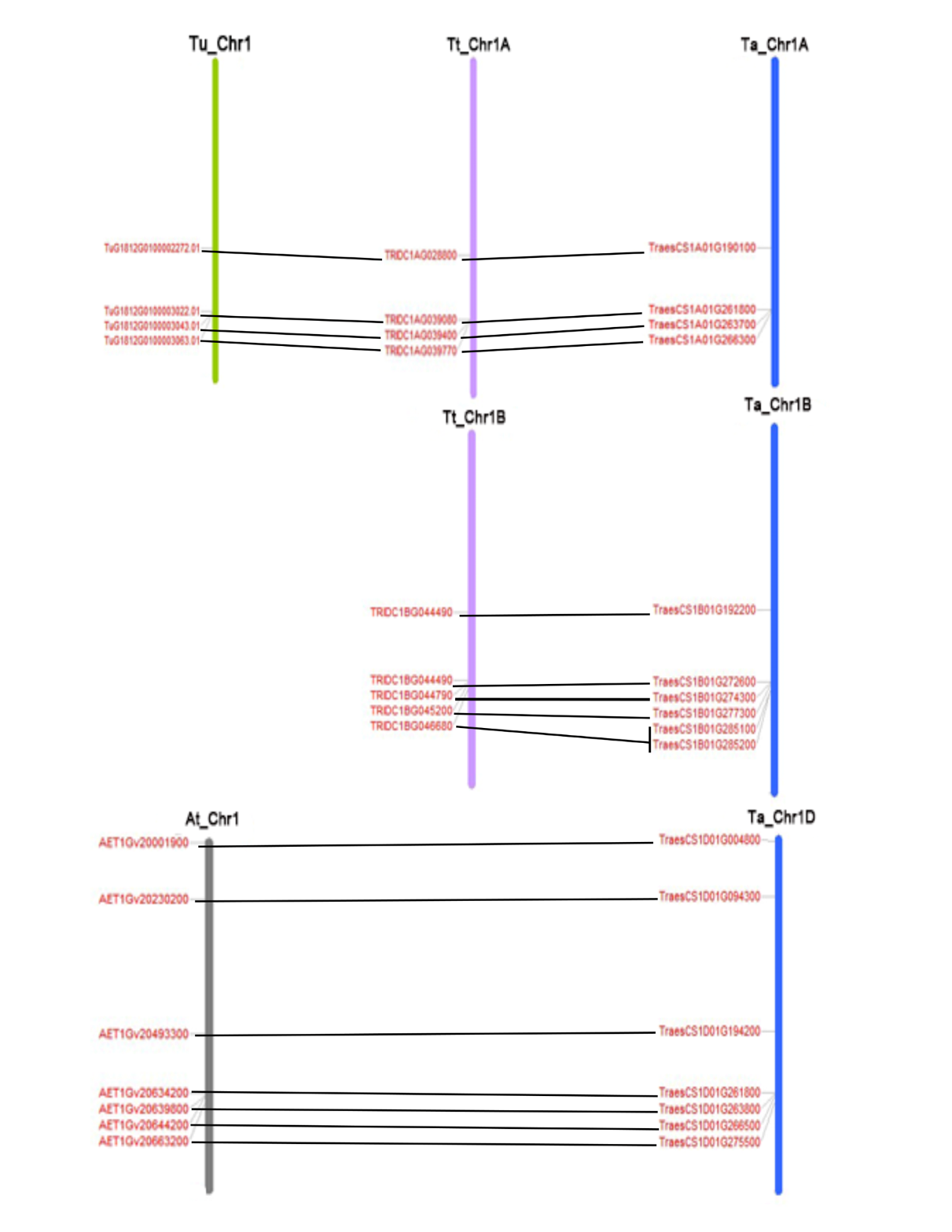
**

**Figure S1.** The evolutionary relationship of NAC transcription factors in Chr 1 between *Triticum urartu* (Tu)*, Aegilops tauschii* (At), *Triticum turgidum* (Tt) and hexaploid wheat (Ta).

**
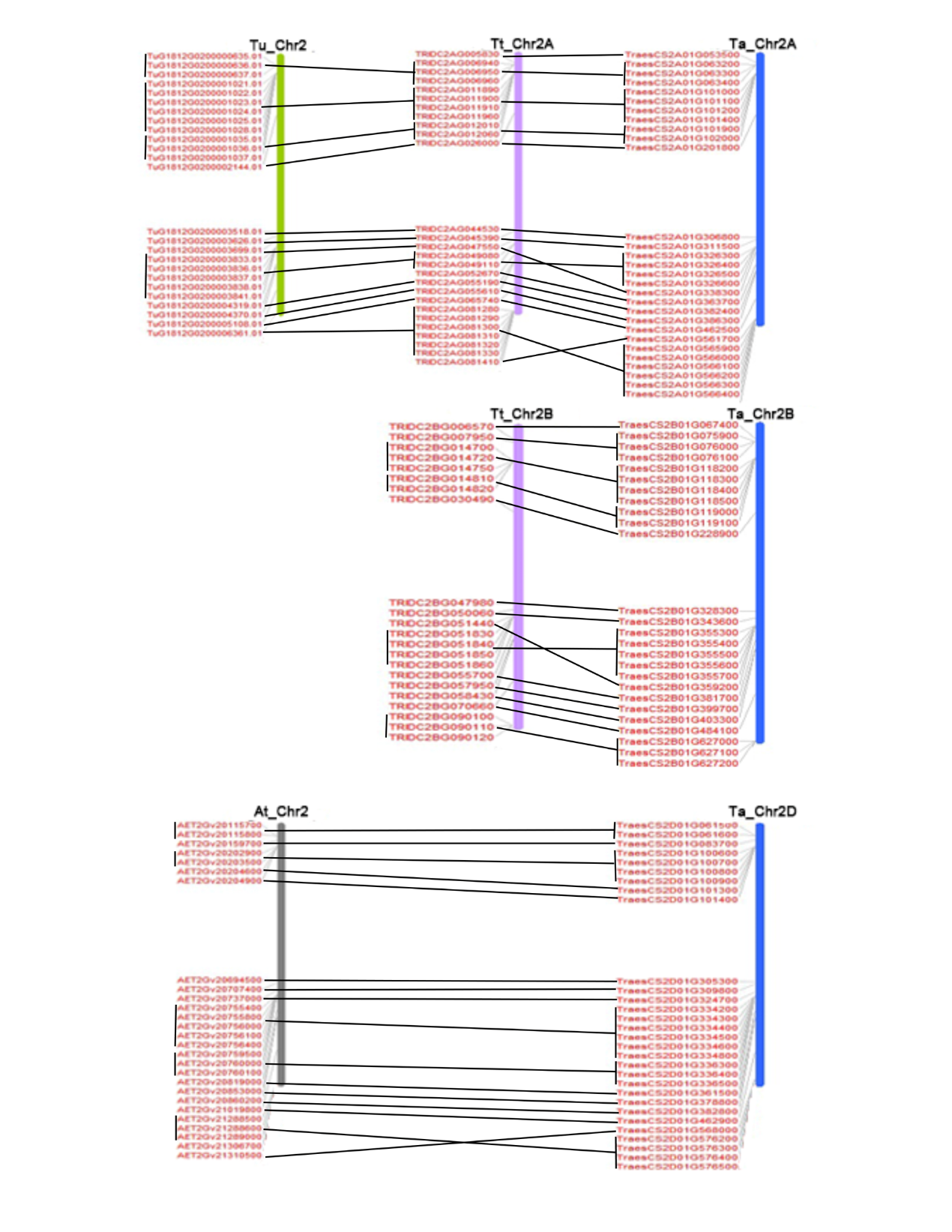
**

**Figure S2.** The evolutionary relationship of NAC transcription factors in Chr 2 between *Triticum urartu* (Tu)*, Aegilops tauschii* (At), *Triticum turgidum* (Tt) and hexaploid wheat (Ta).

**
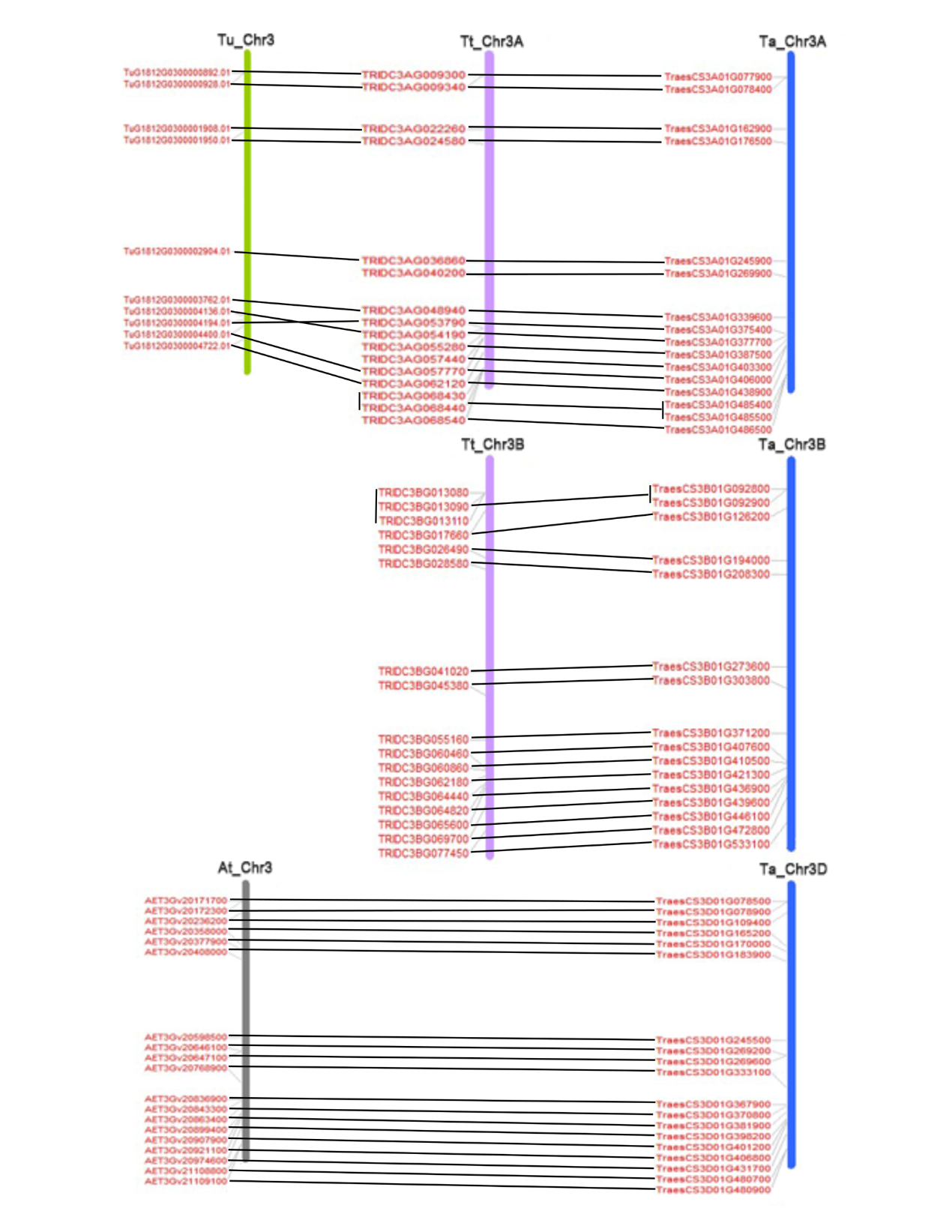
**

**Figure S3.** The evolutionary relationship of NAC transcription factors in Chr 3 between *Triticum urartu* (Tu)*, Aegilops tauschii* (At), *Triticum turgidum* (Tt) and hexaploid wheat (Ta).


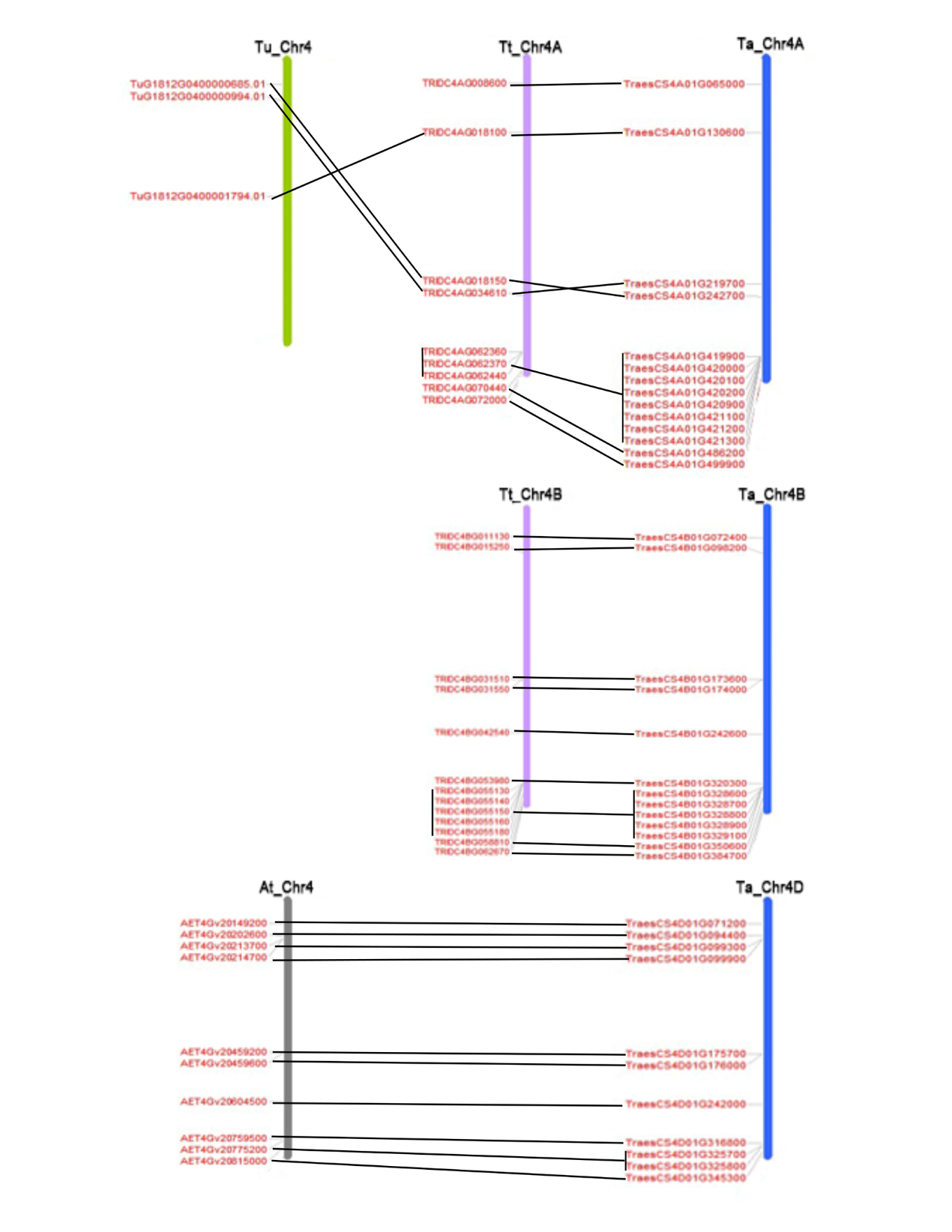


**Figure S4.** The evolutionary relationship of NAC transcription factors in Chr 4 between *Triticum urartu* (Tu)*, Aegilops tauschii* (At), *Triticum turgidum* (Tt) and hexaploid wheat (Ta).


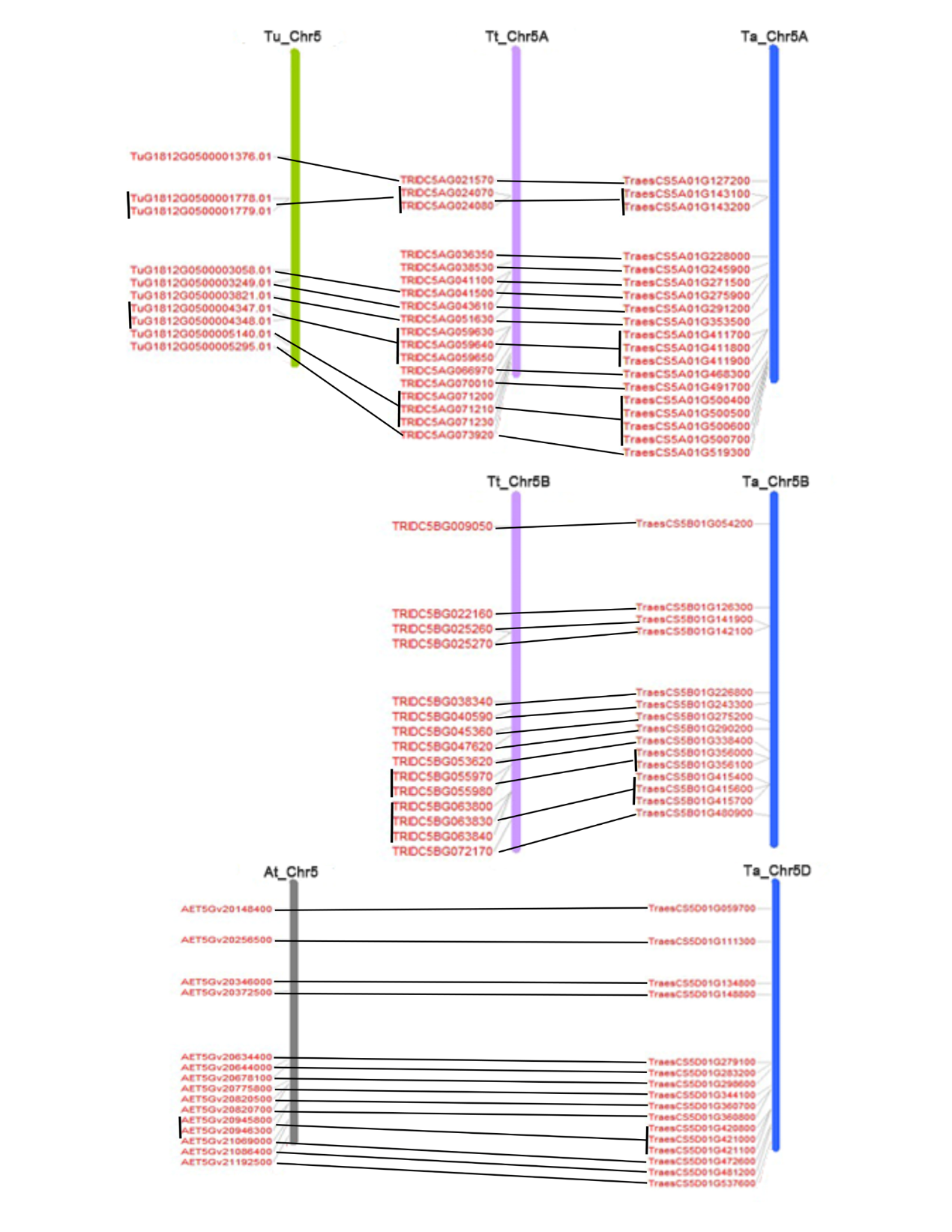


**Figure S5.** The evolutionary relationship of NAC transcription factors in Chr 5 between *Triticum urartu* (Tu)*, Aegilops tauschii* (At), *Triticum turgidum* (Tt) and hexaploid wheat (Ta).


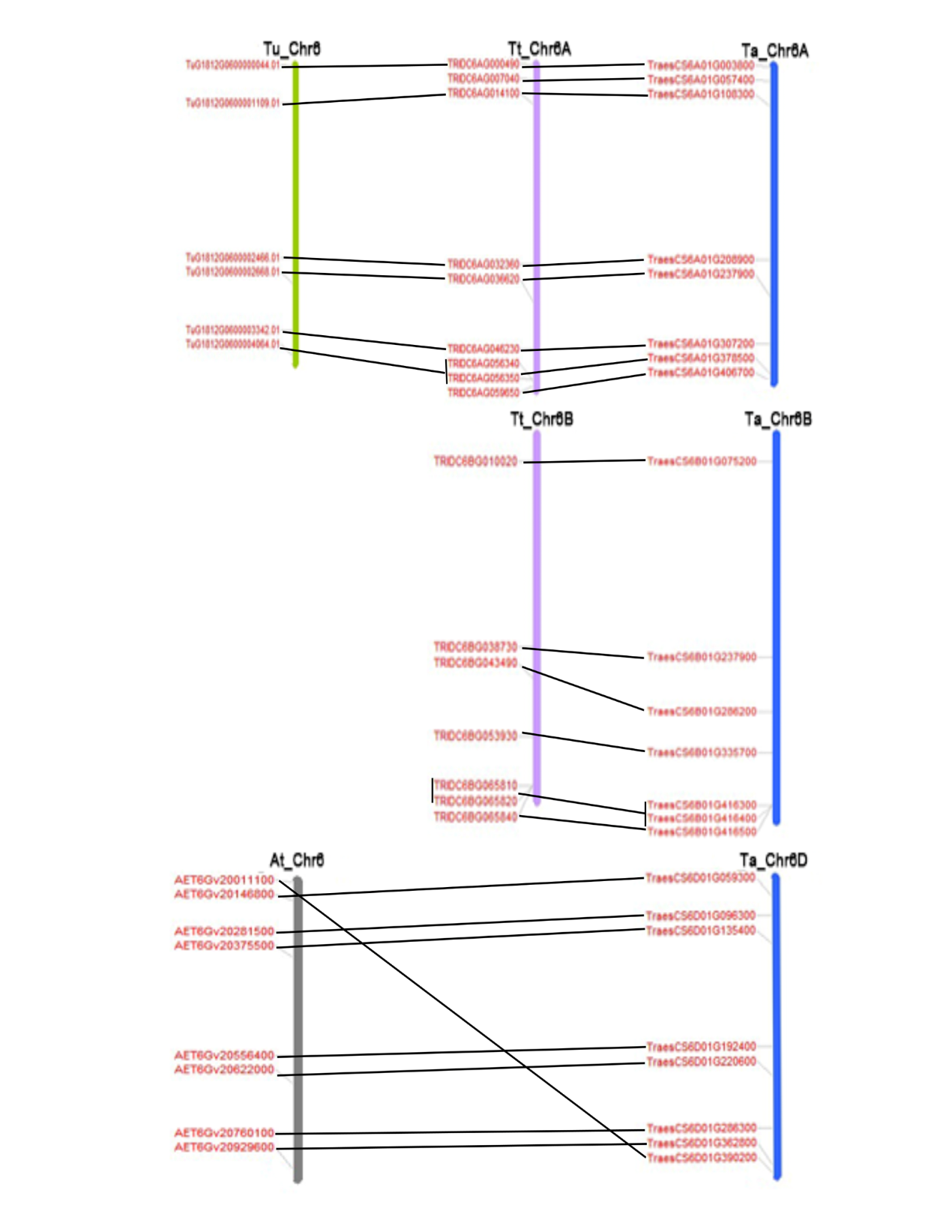


**Figure S6.** The evolutionary relationship of NAC transcription factors in Chr 6 between *Triticum urartu* (Tu)*, Aegilops tauschii* (At), *Triticum turgidum* (Tt) and hexaploid wheat (Ta).


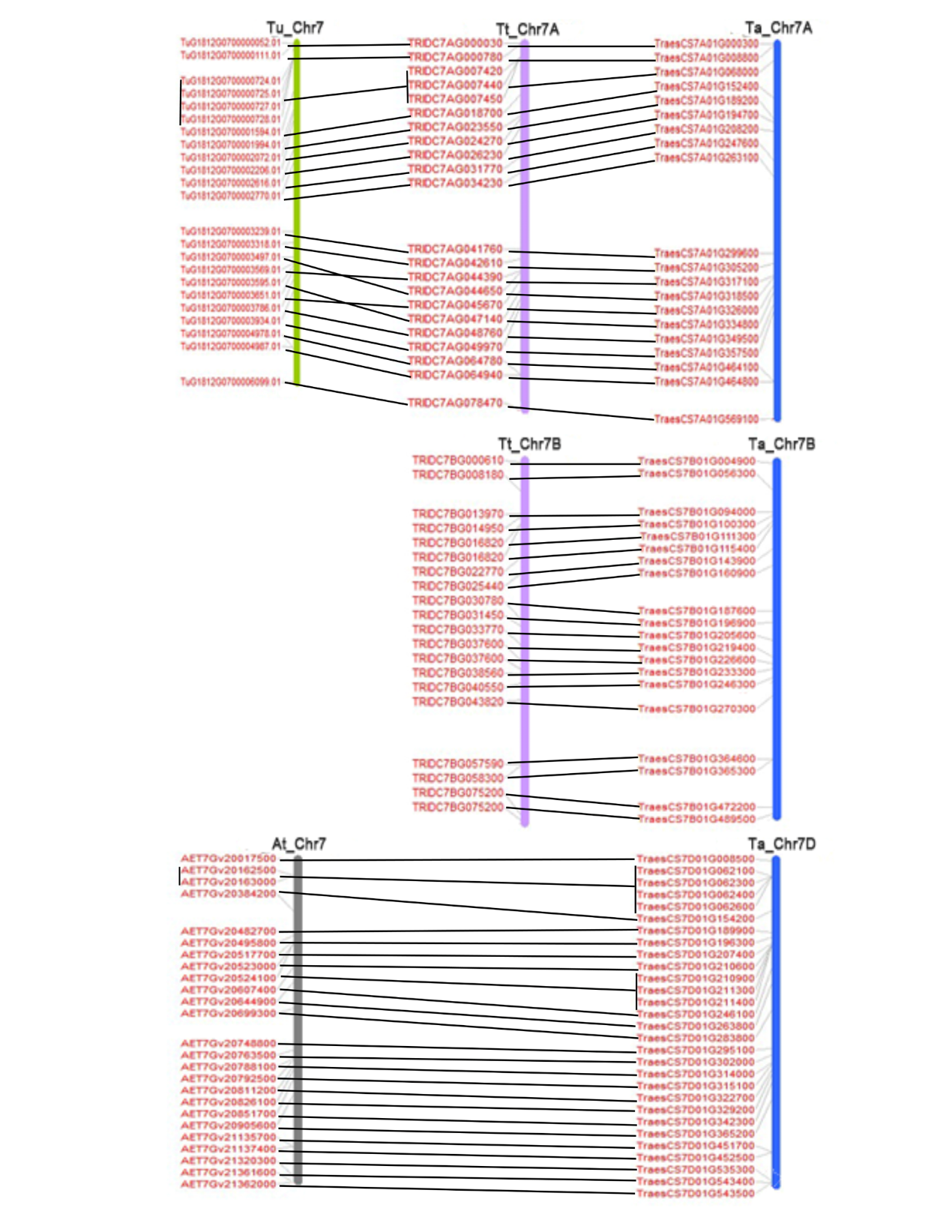


**Figure S7.** The evolutionary relationship of NAC transcription factors in Chr 7 between *Triticum urartu* (Tu)*, Aegilops tauschii* (At), *Triticum turgidum* (Tt) and hexaploid wheat (Ta).

**
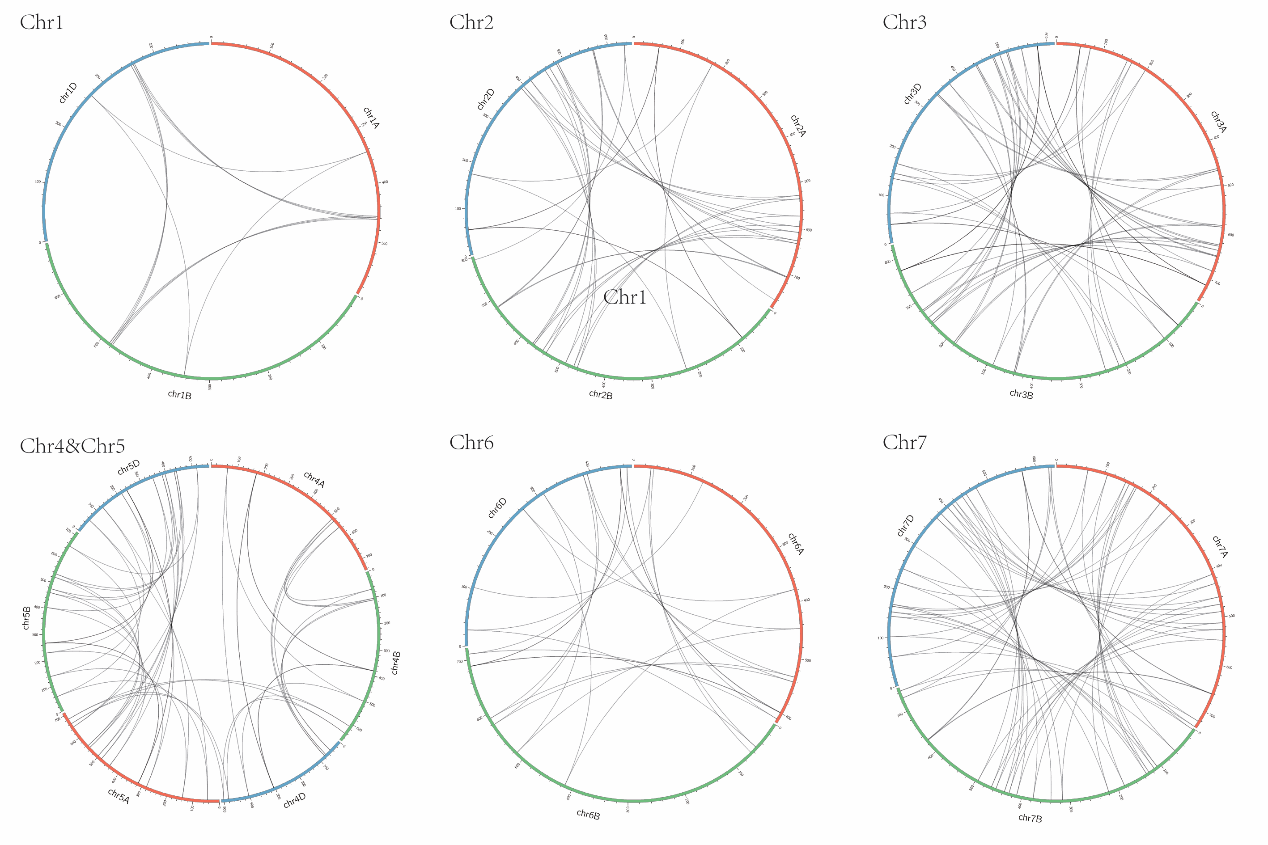
**

**Figure S8.** Circos diagram of TaNACs from chromosome 1 to chromosome 7 of bread wheat.
